# The Rise in Cardiovascular Risk Factors and Chronic Diseases in Guyana: A Narrative Review

**DOI:** 10.5334/aogh.3060

**Published:** 2021-05-31

**Authors:** Loshana Sockalingam, Dipika Desai, Arthur Wong, Gangji Azim, Budhendranauth Doobay, Zahira Khalid, Sonia S. Anand

**Affiliations:** 1 Medical Sciences Graduate Program, Faculty of Health Sciences, McMaster University, Hamilton, ON, Canada; 2 Population Health Research Institute, Hamilton, ON, Canada; 3 Hamilton Health Sciences Corporation, Hamilton, ON, Canada; 4 Division of General Internal Medicine, Department of Medicine, McMaster University, Hamilton, ON, Canada; 5 Division of Nephrology, Department of Medicine, McMaster University, Hamilton, ON, Canada; 6 Nocturnal Hemodialysis Program, St. Joseph’s Hospital, Hamilton, ON, Canada; 7 Doobay Medical Research Centre, Anandale, Guyana; 8 Division of Cardiology, Department of Medicine, McMaster University, Hamilton, ON, Canada; 9 Department of Health Research Methods, Evidence and Impact, Faculty of Health Sciences, McMaster University, Hamilton, ON, Canada

## Abstract

**Background:**

Guyana experiences health challenges related to both communicable and non-communicable diseases. Cardiovascular disease (CVD) is the most common non-communicable disease in Guyana. The main causes of the increased prevalence of non-communicable diseases are modifiable risk factors (e.g. obesity, hypertension, elevated cholesterol, unhealthy dietary patterns) and non-modifiable risk factors (e.g. age and genetics).

**Objective:**

The aim of this review is to understand CVD and risk factor data, in the context of ethnicity in Guyana.

**Methods:**

A review of the published literature as well as government and international health agency reports was conducted. All publications from 2002–2018 describing CVD and related risk factors in Guyana were screened and extracted.

**Findings:**

The population of Guyana is comprised of six ethnic groups, of which East Indian (39.8%) and African (29.3%) are the majority. CVD accounts for 526 deaths per 100,000 individuals per year. Among Indo-Guyanese and Afro-Guyanese, CVD is the primary cause of death affecting 32.6% and 22.7% of the populations, respectively. Within the Indo-Guyanese and Afro-Guyanese communities there is a high prevalence of hypertension and diabetes among individuals over the age of 50. There is a lack of available data describing ethnic disparities in CVD and related risk factors such as obesity, smoking, alcohol, physical activity and diet in Guyana.

**Conclusions:**

Important knowledge gaps remain in understanding the ethnic disparities of CVD and related risk factors in Guyana. Future research should focus on high risk populations and implement widespread screening and treatment strategies of common risk factors such as hypertension, diabetes, and elevated cholesterol to curb the epidemic of CVD in Guyana.

## Introduction

The burden of non-communicable disease is increasing globally, especially in developing countries such as Guyana, located along the borders of Venezuela, Suriname, and Brazil, in South America [1,2]. Guyana is divided into 10 regions classified as either hinterland (rural) or coastal (urban). The largest proportion of the population lives in the coastland regions (89.1%), especially in Georgetown, the capital of Guyana (41.7%) [3]. Guyana’s population consists of seven heterogenous ethnic groups, with the dominant groups being of East Indian (39.8%) and African (29.3%) origins [3]. Guyana experiences health challenges related to both communicable and non-communicable diseases (NCDs), with the highest burden of morbidity and mortality caused by NCDs such as cardiovascular disease (CVD) [4,5]. The main causes of the increased prevalence of NCDs are modifiable risk factors (e.g., obesity, hypertension, elevated cholesterol, unhealthy dietary patterns) and non-modifiable risk factors (e.g., age and genetics) [6]. The impact of this disease is evident in the prevalence of NCD risk factors and the population attributable risk which varies between ethnic groups within Guyana. Therefore, in this review, existing literature is presented in relation to CVD and risk factors such as diabetes, hypertension and obesity, in the context of ethnicity in Guyana.

## Methods & Sources of Data

This narrative review includes studies by independent researchers and reports by government and international health agencies. The literature review was conducted using Google Scholar, Scholars Portal Journals and PubMed databases. Key words used in this literature search are found in Appendix A. Full text publications were reviewed and analyzed for inclusion. Additional references were identified by a manual search of the reference lists of the selected articles. Articles published between 2000–2018 were included. Additional statistical data from Pan America Health Organization Guyana, World Health Organization, the Ministry of Health Guyana and the Bureau of Statistics Guyana between 2002 and 2016 were reviewed and included. ***Table 1*** lists the definition of key terms used in this review.

**Table 1:** Definition of Key Terms.


**Non-Communicable Diseases**	A heterogenous group of medical conditions or diseases that are non-infectious and non-transmissible [7]

**Cardiovascular Disease**	A heterogenous group of diseases that affect the heart or blood vessels [8]

**Diabetes Mellitus**	A disease in which the body cannot 1) produce insulin or 2) use the produced insulin [9]

**Ischemic Heart Disease**	A condition caused by narrowed heart arteries, leading to a reduction in blood and oxygen supply to the cardiac muscles [10]

**Hypertension**	A condition in which the force of the blood against the walls of the blood vessels is consistently too high [11]

**Cerebrovascular Disease**	A heterogenous group of diseases that affects the blood vessels of the brain as well as blood supply to the brain [12]

**Stroke**	A disease affecting the arteries leading to and within the brain, in which a blood vessel is blocked by a clot or ruptures [13]

**Binge Drinking**	Heavy alcohol usage within a short period of time, leading to a blood alcohol concentration of 0.08g or above [14]

**Myocardial Infarction**	Commonly known as a heart attack; Occurs when a portion of the heart is deprived of blood and oxygen [15]

**Urbanization**	The migration towards a Western environment, which increases the consumption of energy rich foods and decreases energy expenditure [16]


## Results

**Figure 1:**
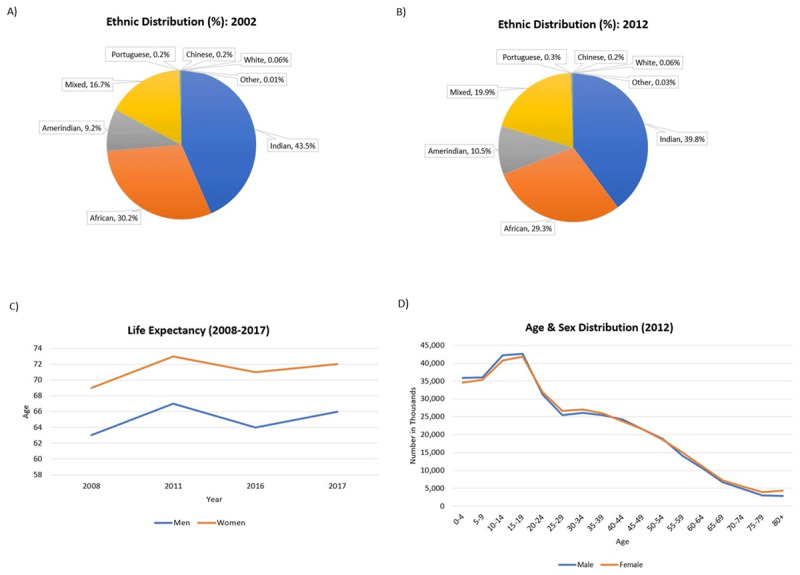
The demographic composition of Guyana **(A)** The ethnic distribution in 2002 **(B)** The ethnic distribution in 2012 **(C)** The life expectancy of men and women from 2008–2017 **(D)** The age and sex distribution in 2012 [1,3,4,17,20,21,22].

The population of Guyana is comprised of six ethnic groups and mixed-race individuals (a result of intermarriage between ethnic groups over time). In 2012, the population consisted of East Indian (39.8%), African (29.3%), mixed-race (19.9%), Amerindians (10.5%), Portuguese (0.3%), Chinese (0.2%), and White (<0.1%) backgrounds (***Figure 1B***). Five ethnic groups resulted from immigration for slavery and indentured labour due to Guyana’s colonial past, while the Amerindians are indigenous to the land [17].

Guyana’s population in 2002 was 751,223, with Indo-Guyanese comprising the majority of the population (43.5%). The country’s population declined by 2012 to 746,955, as a result of emigration [1,3], and declined further by 2017 to 742,300 [8]. However, the distribution of ethnic groups remains unchanged, despite the increase in minority racial groups (***Figure 1A, B***) [3].

In 2008, the life expectancy of women and men was 69 and 63 years, respectively [4]. The life expectancy in 2011 increased to 73 years for women and 67 years for men [20]. In 2016, the life expectancy was 71 years for women and 64 years for men [21]. In 2017, as expected, the life expectancy for women (72 years) was higher than men (66 years) [22]. Regardless of the fluctuations between 2005 to 2017, women consistently live an average 6 years longer than men (***Figure 1C***).

In 2012, there were 375,150 women (50.2%) and 371,805 men (49.8%) in Guyana. Of this, 30% of women and 29% of men were over the age of 20. Over time, the percentage of women over the age of 20 is consistently higher than men (***Figure 1D***) [3]. This may be a result of higher suicide rates (14.2/100,000 women compared to 46.6/100,000 men as of 2016) and traffic accident deaths (74%) observed in men [18,19].

The age-adjusted CVD mortality rate in the Americas was 167.9/100,000 in 2007, and the ratio of ischemic heart disease (IHD) to cerebrovascular deaths is 1.9 (***Table 2***). Noticeably, in the Americas, Guyana has the highest CVD death rate (291.9/100,000) [24], and the ratio of IHD/cerebrovascular disease deaths of 1.2 demonstrates the double burden of IHD and cerebrovascular deaths faced by Guyana compared to other countries in the Americas.

**Table 2:** Mortality rates (age-adjusted rate/100,000) due to CVD, IHD and cerebrovascular disease of selected countries in the Americas [24].


COUNTRY (YEAR)*	CARDIOVASCULAR DISEASE	ISCHEMIC HEART DISEASE	CEREBROVASCULAR DISEASE	RATIO OF IHD/CEREBROVASCULAR DISEASE DEATHS

Region of the Americas (2007)	167.9	71.7	37.3	1.9

Guyana (2006)	291.9	104.4	87.6	1.2

Puerto Rico (2007)	121.0	57.9	24.8	2.3

Trinidad & Tobago (2007)	288.5	128.5	77.8	1.6

Cuba (2009)	205.4	93.0	55.8	1.7

Suriname (2007)	215.3	62.9	99.4	0.6

Venezuela	246.1	123.8	64.1	2.0


* Latest year available for each country

**Figure 2:**
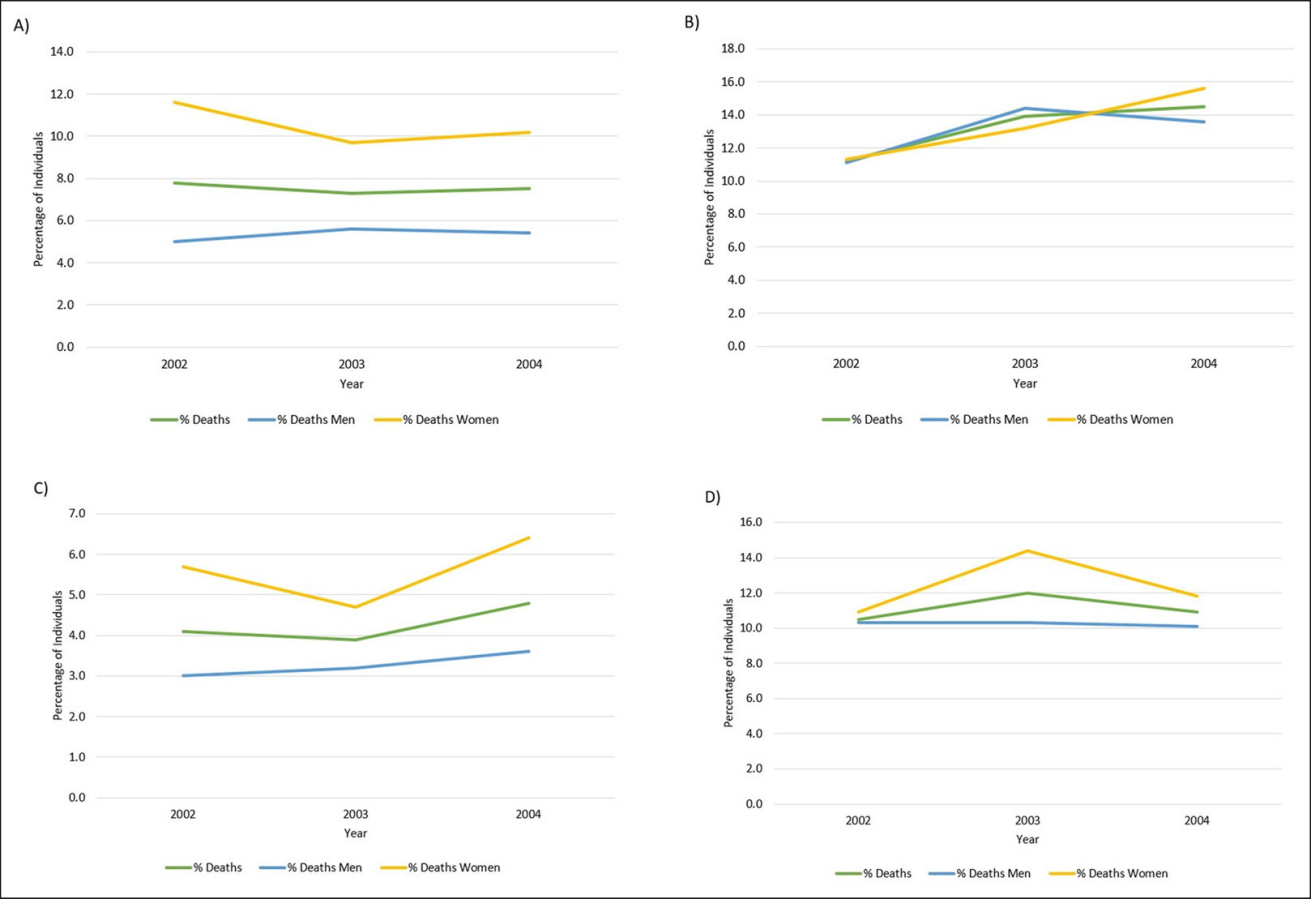
The primary causes of death in relation to sex in Guyana from 2002 to 2004 **(A)** Diabetes Mellitus **(B)** Ischemic and Other Heart Disease **(C)** Hypertension **(D)** Cerebrovascular Disease [5].

Guyana is experiencing a rise in the burden of NCDs which are the leading cause of death, accounting for 822 deaths per 100,000 individuals per year. CVD, the most common NCD, accounts for 526 deaths per 100,000 individuals per year [23]. In 2017, the leading causes of death in Guyana were IHD and stroke. Since 2007, IHD has increased by 16.6% and stroke has increased by 17.8% [22]. ***Figure 2*** demonstrates that women are twice as likely to die from diabetes than men; similarly, deaths due to hypertension is consistently higher among women, which results in a similar IHD death rate and a higher cerebrovascular death rate among women compared to men [5]. Overall, CVD and CVD-related risk factors are the most significant NCD and modifiable risk factors facing adults in Guyana.

**Figure 3:**
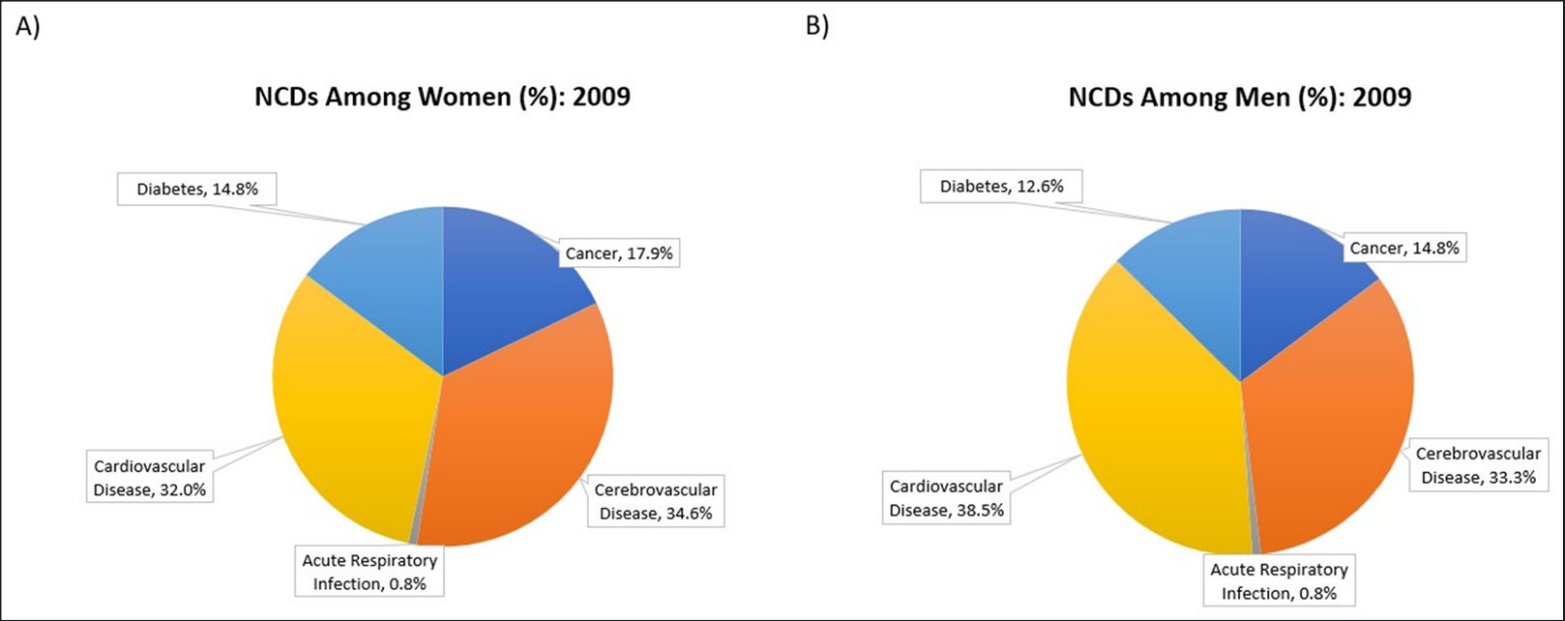
The leading causes of mortality in Guyana in 2009 **(A)** Among women and **(B)** Among men [17].

Cerebrovascular and cardiovascular diseases are the leading causes of mortality in the Indo- and Afro-Guyanese populations, whereas among Amerindians, cancer is the number one cause of death, followed by cerebrovascular disease. The leading cause of mortality among women and men were cerebrovascular disease and cardiovascular disease, respectively (***Figure 3***) [17].

### Trends in CVD Risk Factors

**Figure 4:**
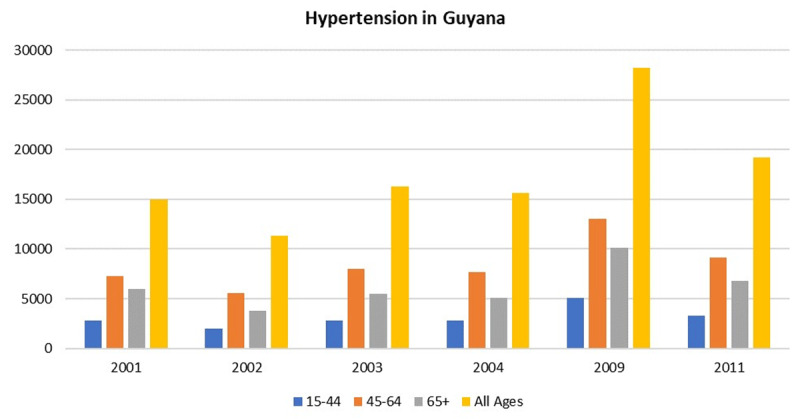
Incidence of hypertension in Guyana by age, 2001–2011 [19].

**Figure 5:**
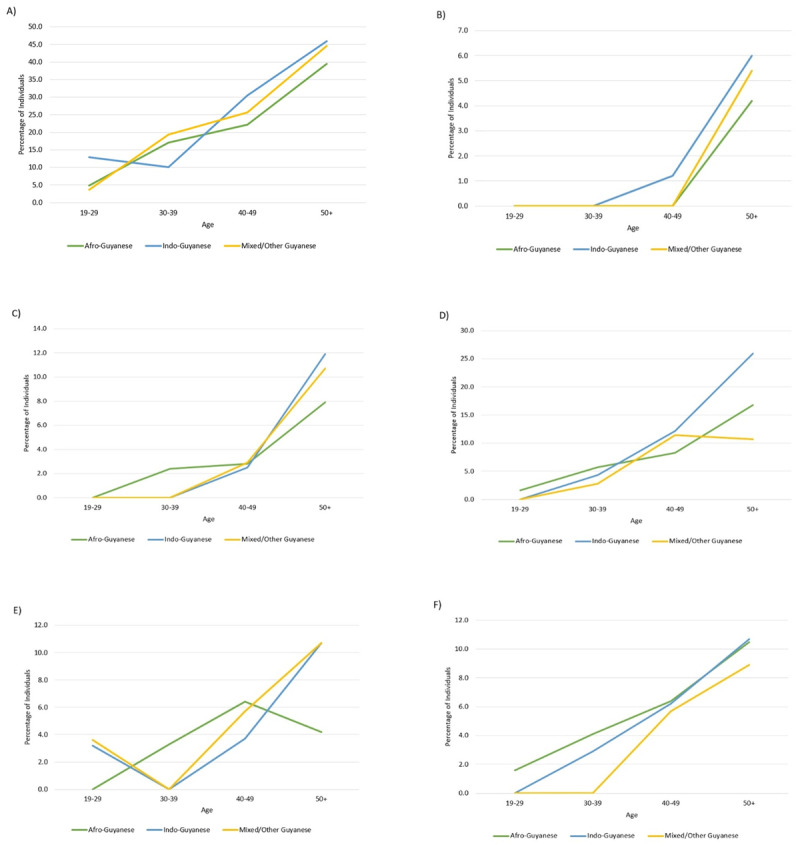
The age specific prevalence of chronic illnesses in Guyana in relation to ethnicity **(A)** Hypertension **(B)** Stroke **(C)** Heart Attack or Heart Problem **(D)** Diabetes Mellitus **(E)** High Cholesterol **(F)** Blood Circulation Problem/Hardening of Arteries [5].

In Guyana, hypertension is the dominant risk factor for IHD and cerebrovascular disease with a population attributable risk (PAR) of 23.4% and 47.9%, respectively [26,27], and is the second leading cause of morbidity [17,25]. There is a substantial burden of hypertension affecting men and women which is especially apparent between the ages of 45–64 years (***Figure 4***) [28]. From 2007 to 2017, hypertensive heart disease increased in prevalence by 32% [22]. Among all ethnicities, hypertension is the most frequently reported chronic illness (46% in Indo-, 45% in mixed-race and 40% in Afro-Guyanese) for individuals over the age of 50 (***Figure 5***) [5].

Type 2 diabetes is also an important risk factor for IHD and stroke with a PAR of 12.3% and 3.9%, respectively [26,27]. In 2012, 64,800 adults (15.13%) were diagnosed with diabetes, yet many remain undiagnosed in Guyana [29]. On average, 2000 new cases of diabetes were diagnosed each year [30]; 74% of individuals diagnosed with diabetes are under the age of 65 years, and most of those are women [5]. In 2017, there was a 10.3% increase in the prevalence of diabetes [22]. Among individuals over the age of 50 years, 25.9% Indo-, 16.8% Afro-Guyanese and 10.7% mixed-race are affected by diabetes. Interestingly, Afro-Guyanese have the highest prevalence of type 2 diabetes in the population under the age of 40, but Indo-Guyanese have a higher prevalence in the population over the age of 40 (***Figure 5***) [5].

Obesity is a risk factor for diabetes and hypertension, and PAR associated with IHD and stroke are 33.7% and 18.6%, respectively [26,27]. In 2010, 11% of women were underweight (BMI <18.5), 26% were overweight (BMI 25.0–29.9) and 22% were obese (BMI >30.0) in Guyana. With increasing educational attainment, the percentage of overweight and obese women decreased; however, with wealth, the opposite effect occurred. Likewise, approximately 12% of men were underweight, 23% were overweight, and 9% were obese. Furthermore, men with a higher level of education and wealth had the highest prevalence of overweight or obese classification. Thus, significantly more women were overweight or obese compared to men [34]. Among both women and men, the highest proportion of being overweight or obese is observed in the 30–49 years of age range. In Guyana, abdominal obesity, a measure of fat distribution, is not extensively measured nor reported in the literature.

Smoking is a behavioural risk factor for IHD and stroke with a PAR of 36.4% and 12.4%, respectively [26,27]. The 2009 Guyana Demographic and Health Survey measured the prevalence of smoking between ages 15–49 years. Smoking is far less common among women than men, with only 3% of women using cigarettes, in contrast to 29% of men. With age, the percentage of women and men who smoke increases [34]. Urban women have a higher percentage of smoking (5%) compared to rural women (2%), whereas urban men smoke less (23%) compared to rural men (32%). Education and wealth did not have a significant impact on smoking in women, however, men with secondary school or higher education in the highest wealth category are the least likely to smoke [34]. In 2013, 15% of the Guyanese population were smokers, with significantly more men (35%) than women (4%) [25].

Alcohol is a behavioural risk factor for IHD and stroke with a PAR of 13.9% and 5.8%, respectively. Moderate alcohol intake is associated with a reduced risk of myocardial infarction; however, binge drinking increases the risk of myocardial infarction and stroke [26,27]. In 2010, the prevalence of binge drinking in Guyana was 6.1% overall, significantly higher among men than women (10.7% vs. 1.7%) [35]. In 2017, alcohol use was ranked 6th in the top 10 risks contributing to disability-adjusted life years [22].

Lack of physical activity is a risk factor for IHD and stroke with a PAR of 25.5% and 35.8%, respectively [26,27]. The Gender Analysis of Selected Non-Communicable Diseases in Guyana (2013) study showed that men are more physically active. Participants reported that cultural factors play a role, as young girls are encouraged to stay indoors to play with dolls, cook and sew while young boys are told to play outdoors. However, the gender differences in physical activity levels also vary by region. For example, in the rural communities, men and women have similar physical activity patterns due to the lack of transportation, unreliable electricity and agriculture equipment [17]. Prevalence and ethnic-specific physical activity rates in Guyana are not extensively reported in the literature.

Diet is a risk factor for IHD and stroke with a PAR of 12.9% and 23.2%, respectively [26,27]. Some components of dietary intake are associated with myocardial infarction and stroke. For example, fruit, vegetable, and legume consumption are associated with a lower risk of CVD, whereas refined grains and high carbohydrate intake are associated with an increased risk of CVD mortality [36,37]. In an older study conducted in 1970, Ashcroft et al. stated that a typical Guyanese diet primarily consisted of starch (rice, yams, sweet potatoes, breadfruit, plantains, corn flour and wheat flour). Protein intake was lower, sugar consumption was moderate and the main fat used in cooking was coconut oil. However, differences existed depending on an individual’s ethnicity. For instance, Afro-Guyanese individuals consumed more rice, ground provisions (yams and cassava), and coconut. Indo-Guyanese ate more flour, peas and spices. The Amerindians ate more cassava, beef and beans, and local fruit and vegetables [38]. More recently, Guyana has shifted away from traditional diets based on home-grown produce to a more energy-dense diet based on processed foods and beverages, known as the Western diet. The food consumption pattern depicts the lifestyle and habits of developed countries, in which foods tend to be animal based, and contain more sugar and fats [17]. Also, no recent investigations of diet patterns using food frequency questionnaires, or 24-hour recalls were identified and studies of diet by ethnicity are lacking. It is likely that the Westernization of the Guyanese diet is in part influencing the rise in CVD risk factors and CVD.

### Access to Health Care

Disparities exist between the urban centers and rural communities. In the urban centers, the public has easy access to hospitals, however, in rural areas, many communities have little or no access to physician-based services or hospital care; [39,40] 12.5% of Guyana’s population does not have access to any form of health care [40]. Furthermore, the health care institutions are not equally distributed across Guyana. Public and private hospitals are located in Georgetown, while the remaining urban centers have regional hospitals. In contrast, the health facilities of the rural regions are basic and limited to health posts and centers [39]. As a consequence of the discrepancy in health service access, the hinterland residences have an increased vulnerability to NCD morbidity and mortality. Moreover, there is a shortage of health care professionals which further reduces the access to health care. In 2005, it was reported that the regional average was 20.4 physicians, and 71.5 nurses and midwives per 10,000 individuals [41].

## Discussion

**Figure 6:**
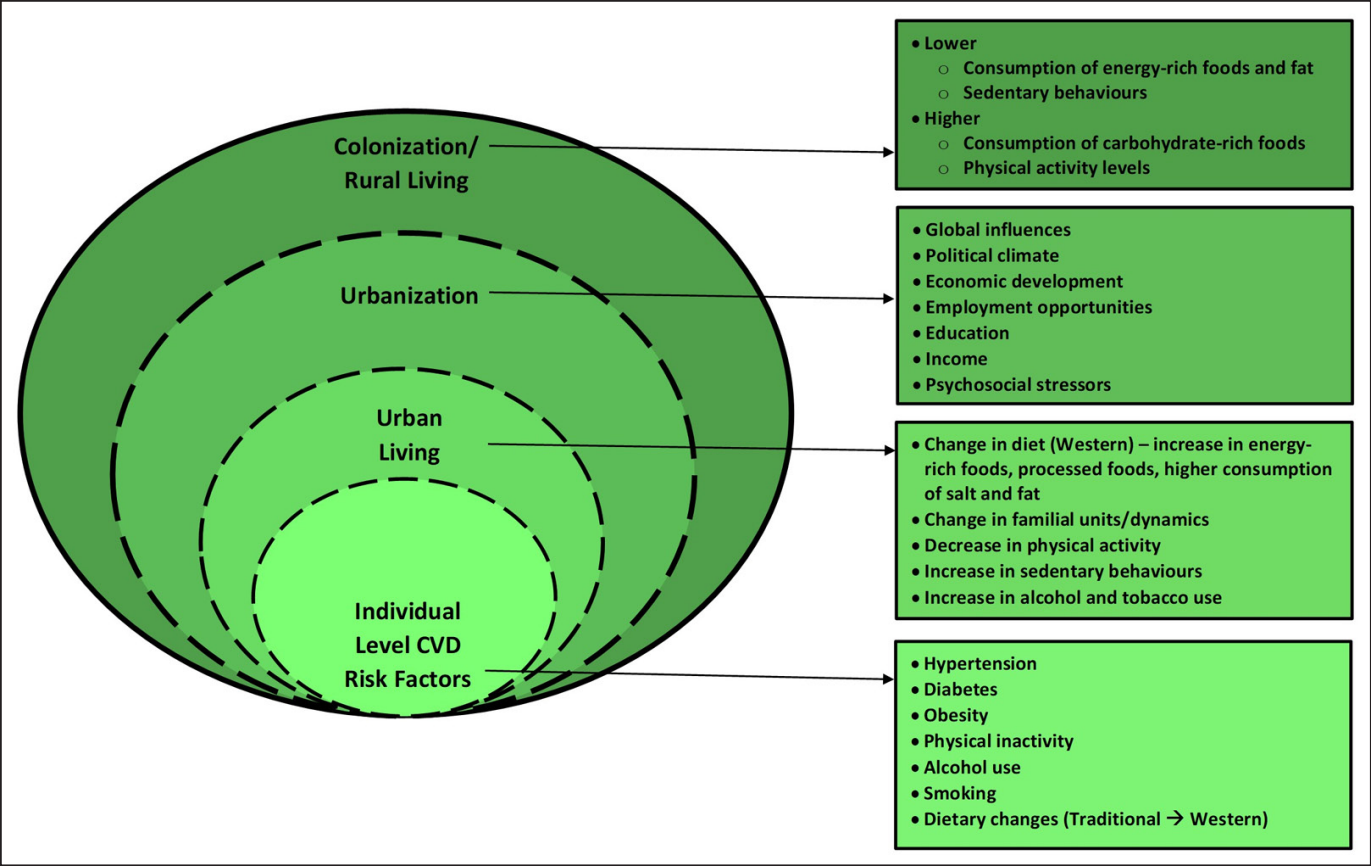
Pathway for the development of cardiovascular disease in Guyana [31,32]. Situated in the broader context of urbanization, the figure describes the broad social and individual-level contributors to the development of CVD in Guyana.

Guyana has followed the typical epidemiological transition resulting in a high burden of CVD risk factors. The epidemiological transition refers to the shift from nutritional deficiencies and infectious diseases to degenerative diseases (CVD, cancer and diabetes) [16]. According to Yusuf et al. (Part I, 2001), there are five stages to the epidemiologic transition for CVD: 1 – Age of pestilence and famine; 2 – Age of receding pandemics; 3 – Age of degenerative and man-made diseases; 4 – Age of delayed degenerative diseases; and 5 – Age of health regression and social upheaval. Guyana is in the midst of stages 2 and 3. During stage 2, the burden of infectious disease is reduced, energy intake is improved, and hypertension increases in prevalence. In stage 3, life expectancy is improved, and a high-fat diet, cigarette smoking, and a sedentary lifestyle become common. NCDs become more prevalent in the population, with CVD accounting for the highest mortality. Concurrently, global influences are impacting local lifestyles towards a more Western way of life. The high burden of CVD in Guyana is attributed to the increase in urbanization and changes to lifestyle (diet, physical inactivity, alcohol, and drug use), which increases the exposure to risk factors (diabetes, obesity, hypertension) as seen in ***Figure 6*** [16]. Prevention strategies to decrease energy intake and increase regular physical activity, to minimize tobacco and alcohol use, and to maintain a balanced diet (high in fruits and vegetables and low in salt and fats) are needed to prevent obesity, diabetes, and hypertension [42,43,46].

The government of Guyana has implemented population-based health programs such as the Guyana Nutrition Strategy 2011–2015, and the National Strategy for Prevention and Control of Chronic Diseases. These programs aim to reduce risk factors, integrate disease management, increase surveillance and improve public policy [44]. As a result, key achievements have been made in an effort to reduce CVD and related risk factors in the population such as smoke free indoor public places, a multi-sector food and nutrition plan and ongoing physical activity campaigns [45]. Population-based health promotions and risk awareness campaigns (i.e., mass media advertisements, brochures and pamphlets) have been utilized to change risk factor promoting behaviours [44]. However, there is still a great need for effective screening and treatment programs.

Health care professionals in Guyana are overburdened and the shortage of physicians is pronounced. Another strategy for CVD prevention which has been used successfully in other countries is the use of non-physician health workers (NPHW) to screen and implement treatment of CVD risk factors. The effectiveness of this strategy was demonstrated in the HOPE-4 trial. HOPE-4 [47] was an open, community-based, cluster-randomized controlled trial (n = 1371) in Colombia and Malaysia which included people with new or poorly controlled hypertension. For the 14 communities randomized to the intervention group (n = 644), NPHW treated CVD risk factors using computer-based algorithms and counselling programmes as well as recommended antihypertensive and statin medications under the supervision of a physician, while the 16 control group communities received information only. HOPE-4 reported that this comprehensive model of care led by NPHW improved blood pressure control (69% in the intervention group vs 30% in the control group; p < 0.0001) and CVD risk (a reduction in the Framingham Risk Score for 10 year CVD risk was –11.2% in the intervention vs –6.4% in the control, P < 0.0001) [47]. Therefore, given Guyana’s current status, the use of NPHW represents a promising strategy to reduce the CVD burden in this low-middle income country.

Based on the available data, future observational research should focus on specific groups such as populations inhabiting the rural regions, and the highest risk ethnic groups [17]. Various socioeconomic, dietary, genetic, or environmental factors may affect the ethnic distribution of CVD and its risk factors [2]. In Guyana, official statistics provide little information about health and disease for each ethnic group [5]. Ethnic information is useful to help focus prevention and screening programs. Lastly, statistics should be collected regularly and continually to detect trends over time by age, sex and ethnicity. Sharing available data and reports publicly should be encouraged to facilitate change and improve Guyana’s NCD trajectory [17].

## Limitations

Data on CVD and related risk factors, differentiated by gender, age and ethnicity is limited. Furthermore, some of this data is not publicly available and is out-dated. The latest available census is from the 2012 PAHO, MOH and WHO reports, with limited data from 2017. Data on the incidence and prevalence of CVD within ethnic-specific cohorts is scarce. Moreover, the incidence and ethnic-specific rates of obesity, smoking, alcohol use and physical activity as well as the ethnic specific discrepancies in the access to health care in Guyana are not extensively reported in the literature. Given the nature of Guyana’s ethnic makeup, studying each ethnic group is crucial for disease prevention and treatment strategies.

## Conclusion

CVD is increasing in Guyana due to a high burden of poverty and the presence of a higher number of CVD risk factors (such as elevated cholesterol, diabetes, and diet) [48]. There are variations in the seven risk factors examined (hypertension, diabetes, obesity, tobacco use, alcohol use, physical inactivity and diet) based on ethnic groups. However, more research is needed to fully understand the extent and depth of the ethnic disparities in relation to health outcomes. Unless effective preventive strategies are implemented, the incidence of CVD and its related risk factors will likely continue to rise [33]. These strategies include education regarding healthy active living, early screening, and treatment of risk factors by health care professionals, as well as improving access to cardiac testing and cardiac interventions.

## Appendix A: Summary of search terms, search limiters, databases, and inclusion and exclusion criteria for published literature search

**Table:**

**Key Search Terms**	Population, mortality, cardiovascular disease, type 2 diabetes, diabetes mellitus, hypertension, obesity, overweight, diet, nutrition, physical activity, smoking, alcohol, ethnicity, race, health risks, risk factors, health, South Asian, Indian, African, Amerindians, mixed race, Portuguese, Chinese, Whites, Indo-Guyanese, Afro-Guyanese, Indigenous, heart disease, life expectancy, Guyana, non-communicable disease, prevalence, incidence, health outcomes, burden of disease, trends, morbidity, government statistics, screening programs, treatment programs, primary prevention, health promotion

**Search Limiters**	Country: Guyana Language: English Publication Date: last 18 years (2000–2018)

**Databases**	Scholars Portal Journals, PubMed and Google Scholar

**Inclusion Criteria**	(1) From 2000–2018 (2) Written in English (3) Abstracts that contain one or more of the key search terms identified (4) Specific to the Guyanese population

**Exclusion Criteria**	(1) Unpublished dissertations, editorials, book chapters or papers published before 2000 (2) Content redundant to another selected article

## References

[1] Danns GK. The impact of identity, ethnicity and class on Guyana’s strategic culture. Am Int J Contemp Res. 2014; 4(11): 65–77. http://www.aijcrnet.com/journals/Vol_4_No_11_November_2014/8.pdf.

[2] Plummer WS, Persaud P, Layne PJ. Ethnicity and cancer in Guyana, South America. Infect Agent Cancer. 2009; 4(1): 1–4. DOI: 10.1186/1750-9378-4-S1-S7 19208212 PMC2638466

[3] Bureau of Statistics, Guyana. Compendium 2: Population Composition. 2016; 1–47. Georgetown, Guyana. http://www.statisticsguyana.gov.gy/census.html.

[4] Pan American Health Organization, Guyana. Health in the Americas. 2012; 1–22. https://www.paho.org/salud-en-las-americas-2012/index.php?option=com_docman&view=download&category_slug=hia-2012-regional-volume-19&alias=155-chapter-1-a-century-public-health-americas-155&Itemid=231&lang=en.

[5] Wilson LC, Wilson CM, Johnson BM. Race and health in Guyana: An empirical assessment from survey data. Caribbean Studies. 2010; 38(1): 37–58. DOI: 10.1353/crb.2010.0035 21553433

[6] Krishnadath IS, Venrooij LM, Jaddow VW, et al. Ethnic differences in prediabetes and diabetes in the Suriname health study. BMJ Open Diabetes Res Care. 2016; 4(1): 1–11. DOI: 10.1136/bmjdrc-2015-000186 PMC4932318 27403324

[7] Kim HC, Oh SM. Noncommunicable diseases: Current status of major modifiable risk factors in Korea. Journal of Preventive Medicine & Public Health. 2013; 46(4): 165–172. DOI: 10.3961/jpmph.2013.46.4.165 23946874 PMC3740221

[8] National Heart, Lung and Blood Institute. Know the differences: Cardiovascular disease, heart … (n.d.). Retrieved from https://www.nhlbi.nih.gov/sites/default/files/media/docs/Fact_Sheet_Know_Diff_Design.508_pdf.pdf.

[9] Diabetes Canada. What is diabetes? (n.d.). Retrieved from https://www.diabetes.ca/diabetes-basics/what-is-diabetes.

[10] American Heart Association. Silent ischemia and ischemic heart disease. 2015, 7 31. Retrieved from https://www.heart.org/en/health-topics/heart-attack/about-heart-attacks/silent-ischemia-and-ischemic-heart-disease.

[11] American Heart Association. What is high blood pressure? 2016, 10 31. Retrieved from https://www.heart.org/en/health-topics/high-blood-pressure/the-facts-about-high-blood-pressure/what-is-high-blood-pressure.

[12] Good DC. Cerebrovascular Disease. In: Walker HK, Hall WD, Hurst JW, (eds.), Clinical Methods: The History, Physical, and Laboratory Examinations. 3rd edition. Boston: Butterworths; 1990. Chapter 55. Available from: https://www.ncbi.nlm.nih.gov/books/NBK378/. 21250045

[13] American Heart Association. About stroke. (n.d.). Retrieved from https://www.stroke.org/en/about-stroke.

[14] Ministry of Public Security. Guyana National Household Drug Prevalence Survey Report 2016. Organization of American States/Inter-American Drug Abuse Control Commission (OAS/CICAD); 2016. http://www.cicad.oas.org/oid/pubs/GuyanaHouseholdDrugReport2016.pdf.

[15] Johns Hopkins Medicine. Conditions We Treat: Myocardial Infarction: Johns Hopkins Heart and Vascular Institute. (2017, 6 2). Retrieved from https://www.hopkinsmedicine.org/heart_vascular_institute/conditions_treatments/conditions/myocardial_infarction.html.

[16] Yusuf S, Reddy S, Ounpuu S, et al. Global burden of cardiovascular diseases. Part I: General considerations, the epidemiologic transition, risk factors and impact of urbanization. Circulation. 2001; 104(22): 2746–2753. DOI: 10.1161/hc4601.099487 11723030

[17] Pan American Health Organization & World Health Organization. Gender Analysis of Selected Non-Communicable Diseases in Guyana; 2013. http://www.social-solutions.net/heemskerk/images/Gender_and_NCDs_rapport.pdfHO).

[18] World Health Organization. World Health Organization – Global Health Observatory data repository. Suicide rate estimates, age-standardized Estimates by country – Guyana. 2018; 1. http://apps.who.int/gho/data/node.main.MHSUICIDEASDR?lang=en.

[19] McWade CM, McWade MA, Quistberg DA, et al. Epidemiology and mapping of serious and fatal road traffic injuries in Guyana: Results from a cross-sectional study. Injury Prevention. 2017; 23(5): 303–308. DOI: 10.1136/injuryprev-2016-042119 28947529

[20] Centers for Disease Control and Prevention. Global Health – Guyana; 2014 https://www.cdc.gov/globalhealth/countries/guyana/.

[21] Institute for Health Metrics and Evaluation. Guyana. 2016; 1–8. http://www.healthdata.org/guyana.

[22] Institute for Health Metrics and Evaluation. Guyana. 2017; 1–8. http://www.healthdata.org/guyana.

[23] Dyal N, Dolovich L. Assessment of a hypertension screening and education intervention in Charlestown, Guyana. Can Pharm J (Ott). 2015; 149(1): 46–53. DOI: 10.1177/1715163515617486 PMC4713890 26798377

[24] de Souza MFM, Gawryszewski VP, Orduñez P, et al. Cardiovascular disease mortality in the Americas: Current trends and disparities. Heart. 2012; 98(16): 1207–1212. DOI: 10.1136/heartjnl-2012-301828 22826558

[25] World Health Organization, Guyana. Guyana: World Health Organization – Noncommunicable Diseases (NCD) Country Profiles. 2014; 1. https://www.who.int/nmh/countries/2014/guy_en.pdf.

[26] Yusuf S, Hawken S, Ounpuu S, et al. Effect of potentially modifiable risk factors associated with myocardial infarction in 52 countries (the INTERHEART study): Case-control study. The Lancet. 2004; 364(9438): 937–952. DOI: 10.1016/S0140-6736(04)17018-9 15364185

[27] O’Donnell MJ, Chin SL, Rangarajan S, et al. Global and regional effects of potentially modifiable risk factors associated with acute stroke in 32 countries (INTERSTROKE): A case-control study. The Lancet. 2016; 388(10046): 761–775. DOI: 10.1016/S0140-6736(16)30506-2 27431356

[28] Ministry of Health, Guyana. Guyana Strategic Plan for the Integrated Prevention and Control of Chronic Non-Communicable Diseases and their Risk Factors 2013–2020. 2013; 1–96. Georgetown, Guyana: PAHO. https://www.paho.org/guy/index.php?option=com_docman&view=download&category_slug=not-communicable&alias=119-guyana-ncds-strategic-plan-2013-2020&Itemid=291.

[29] World Health Organization. Guyana: World Health Organization – Diabetes Country Profiles. 2016; 1. http://www.who.int/diabetes/country-profiles/guy_en.pdf.

[30] Pan American Health Organization. Guyana Country Cooperation Strategy 2016–2020: Strengthening Health Systems to Achieve Universal Health. 2017; 1–58. Georgetown, Guyana: https://www.paho.org/guy/index.php?option=com_docman&view=download&alias=177-final-ccs-december-31-01-2017-final-15-feb-2017&category_slug=data-and-statistics&Itemid=291.

[31] Lowe J, Sibbald RG, Taha NY, et al. The Guyana diabetes and foot care project: A complex quality improvement intervention to decrease diabetes-related major lower extremity amputations and improve diabetes care in a lower-middle-income country. PLOS Med. 2015; 12(4): 1–13. DOI: 10.1371/journal.pmed.1001814 PMC4405371 25898312

[32] Jagessar RC, Kingston S. The status of diabetes in Guyana, its health and synthetic drug treatments. World J Pharm Sci. 2015; 4(7): 149–165. www.wjpps.com/download/article/1435648594.pdf.

[33] Jagessar RC, McFarlane D, Parshram S, et al. The status of obesity in selected areas of coastal Guyana. World J Pharm Sci. 2018; 7(5): 1639–1657. www.wjpps.com/download/article/1526089590.pdf.

[34] Ministry of Health, Bureau of Statistics & ICF Macro. Guyana Demographic and Health Survey. Georgetown, Guyana 2010. https://dhsprogram.com/pubs/pdf/FR232/FR232.pdf.

[35] World Health Organization, Guyana. Guyana: Alcohol Consumption. 2014; 1. https://drinkingage.procon.org/sourcefiles/guyana-drinking-age.pdf.

[36] Miller V, Mente A, Dehghan M, et al. Fruit, vegetable, and legume intake, and cardiovascular disease and deaths in 18 countries (PURE): A prospective cohort study. The Lancet. 2017; 390(10107): 2037–2049. DOI: 10.1016/S0140-6736(17)32253-5 28864331

[37] Dehghan M, Mente A, Zhang X, et al. Associations of fats and carbohydrate intake with cardiovascular disease and mortality in 18 countries from five continents (PURE): A prospective cohort study. The Lancet. 2017; 390(10107): 2050–2062. DOI: 10.1016/S0140-6736(17)32252-3 28864332

[38] Ashcroft MT, Beadnell HMSG, Bell R, et al. Characteristics relevant to cardiovascular disease among adults of African and Indian origin in Guyana. Bulletin of the World Health Organization. 1970; (42): 205–223. https://www.ncbi.nlm.nih.gov/pmc/articles/PMC2427442/. PMC2427442 4246109

[39] Ministry of Public Health (Guyana). Health Vision 2020: A national health strategy for Guyana 2013–2020. Georgetown: MPH; 2013. Available from: http://www.paho.org/guy/index.php?option=com_docman&view=download&category_slug=health-systems-and-services&alias=123-guy-healthvision-2013-2020&Itemid=291.

[40] Pan American Health Organization. Second Edition. 2001, 12 14. Health systems and services profile of Guyana. Pan American Health Organization. Retrieved from http://new.paho.org/hq/dmdocuments/2010/Health_System_Profile-Guyana_2001.pdf.

[41] Misir P, Phil M. Progressive health care reforms in Latin America. Health Care in Guyana. Social Medicine. 2015; 9(1): 36–47. Retrieved from www.socialmedicine.info.

[42] Lowe J, Sibbald RG, Taha NY, et al. The Guyana diabetes and foot care project: Improved diabetic foot evaluation reduces amputation rates by two-thirds in a lower-middle-income country. Int J Endocrinol. 2015; 1–6. DOI: 10.1155/2015/920124 PMC4452302 26089901

[43] Cheng A, Prabhakar C, Kapila V, et al. Hypertension in Guyana: Lessons from a health promotion program. Univ Toronto Med J. 2003; 81(1): 8–11. https://www.researchgate.net/publication/50854951.

[44] Pan American Health Organization and Ministry of Health, Guyana. Strategic Plan 2013–2020: Integrated Prevention and Control of Non-Communicable Disease in Guyana. 2013; 1–98. https://www.paho.org/guy/index.php?option=com_content&view=article&id=207:ncds-strategic-plan&Itemid=289.

[45] Ministry of Public Health, Guyana. Featured Projects: Chronic Disease Control; 2016. Retrieved from https://www.health.gov.gy/index.php/programmes/dct/cncd.

[46] Yusuf S, Reddy S, Ounpuu S, et al. Global Burden of Cardiovascular Diseases. Part II: Variations in cardiovascular disease by specific ethnic groups and geographic regions and prevention strategies. Circulation. 2001; 104(23): 2855–2864. DOI: 10.1161/hc4701.099488 11733407

[47] Schwalm, JD, et al. A community-based comprehensive intervention to reduce cardiovascular risk in hypertension (HOPE 4): A cluster-randomised controlled trial. The Lancet. 2019; 394(10205): 1231–1242. DOI: 10.1016/S0140-6736(19)31949-X 31488369

[48] Pramparo P, Montano CM, Barceló A, et al. Cardiovascular diseases in Latin America and the Caribbean: The present situation. Prevention and Control. 2006; 2(3): 149–157. DOI: 10.1016/j.precon.2007.03.002

